# Silicone Oil Emulsification after Vitrectomy for Rhegmatogenous Retinal Detachment

**DOI:** 10.1155/2020/6940625

**Published:** 2020-02-24

**Authors:** Jian Yu, Yuan Zong, Chunhui Jiang, Haohao Zhu, Guohua Deng, Gezhi Xu

**Affiliations:** ^1^Department of Ophthalmology and Vision Science, Eye and ENT Hospital, Fudan University, Shanghai 200031, China; ^2^Key Laboratory of Myopia of State Health Ministry, Key Laboratory of Visual Impairment and Restoration of Shanghai, Shanghai 200031, China; ^3^NHC Key Laboratory of Myopia (Fudan University), Key Laboratory of Myopia, Chinese Academy of Medical Sciences, Shanghai 200031, China; ^4^Department of Ophthalmology, People's Hospital of Shanghai No. 5, Shanghai 200240, China; ^5^Department of Ophthalmology, Third People's Hospital of Changzhou, Changzhou 213001, China

## Abstract

**Purpose:**

To investigate the characteristics of silicone oil (SO) emulsification after vitrectomy for rhegmatogenous retinal detachment and their possible correlations with clinical factors.

**Methods:**

The first 2 mL of washing out fluid after SO removal was collected, and used for the measurement of the size and number of SO droplets using a Multisizer® 3 Coulter counter (Beckman Coulter, USA). The correlations between SO droplets and clinical factors were analyzed.

**Results:**

A total of 38 patients (23 males, 15 females) who underwent primary PPV with SO injection for RRD and whose retina stayed attached for ≥3 months after SO removal were included in the study. The average number of oil droplets was 1.96 × 10^6^ ± 3.95 × 10^6^/mL (range 0.17 × 10^6^ to 21.71 × 10^6^/ml), and 80.8% (range 64.23%–99.07%) of the droplets were 1-2 *μ*m in diameter. The total number of emulsified SO droplets was not correlated with any clinical factor (all *P* > 0.05). When the emulsified SO droplets were divided into groups by their diameter, multiple linear regression revealed that age was negatively correlated with the numbers of 5–7-*μ*m in diameter. The total number of emulsified SO droplets was not correlated with any clinical factor (all *μ*m in diameter. The total number of emulsified SO droplets was not correlated with any clinical factor (all *P* > 0.05). When the emulsified SO droplets were divided into groups by their diameter, multiple linear regression revealed that age was negatively correlated with the numbers of 5–7-*μ*m in diameter. The total number of emulsified SO droplets was not correlated with any clinical factor (all *μ*m in diameter. The total number of emulsified SO droplets was not correlated with any clinical factor (all *P* > 0.05). When the emulsified SO droplets were divided into groups by their diameter, multiple linear regression revealed that age was negatively correlated with the numbers of 5–7-

**Conclusion:**

Using a Multisizer® Coulter counter, we successfully determined the number and size of SO droplets after emulsification. We found that the number of 5–12-*μ*m-diameter droplets was higher in younger-age patients and was higher in patients using antiglaucoma eyedrops.*μ*m in diameter. The total number of emulsified SO droplets was not correlated with any clinical factor (all

## 1. Introduction

Silicone oil (SO) was first introduced into the field of ophthalmology by Cibis et al. [1] in 1962. Since then, SO has become an important tool for postoperative tamponade in complicated vitreoretinal cases. However, the problem of emulsification soon arose [2] and it became a concern for vitreoretinal surgeons. Emulsification was reported to be correlated with many complications, including glaucoma, [3] inflammation and proliferation, [4] cataract, [5], and keratopathy [6]. Additionally, emulsification was evaluated by using silt lamp or under a microscope, [7, 8] which are highly subjective methods and difficult to quantify. To explore the correlation between emulsification and these complications, a reliable method was needed to quantify the degree of SO emulsification. Recently, Chan et al. [9, 10] studied SO emulsification using a Multisizer® Coulter counter (Beckman Coulter, USA). The system can measure the size of nonconducting particles suspended in a fluid based on Coulter's principle [11]. Coulter counters are used in a variety of industries to measure particles in different solutions [12]. Also, several different types of Coulter counters are used to measure blood cells in clinical settings [13]. Chan et al. [9] used the Multisizer® Coulter counter to measure SO droplets, most of which were <2 *μ*m in diameter. However, none has measured the number of SO droplets. Therefore, in the present study, we used a Multisizer® Coulter counter to determine the characteristics of SO emulsification in terms of the size and the number of droplets in a large series of patients who underwent vitrectomy for rhegmatogenous retinal detachment (RRD). We also investigated the potential correlations of SO droplet size and the number with clinical characteristics.

## 2. Methods

### 2.1. Patients

Patients who underwent primary pars plana vitrectomy (PPV) with SO injection for RRD followed by SO removal at the Eye and ENT Hospital of Fudan University were enrolled in this study. The same surgeon (Chunhui Jiang) performed all PPV and SO removal procedures. Exclusion criteria included history of intraocular disease (other than cataract), age <18 years at the time of primary PPV, cataract surgery within 6 months, or retinal redetachment after SO removal. The study was approved by the Ethics Committee of the Eye and ENT Hospital of Fudan University and adhered to the Declaration of Helsinki. Informed consent was obtained from each patient.

Best-corrected visual acuity (BCVA), intraocular pressure (IOP), and fundus evaluation by slit-lamp microscopy and an indirect ophthalmoscopy lens (Maxfield 84 Diopter; Ocular, USA) were assessed before SO removal and 3 months after SO. Demographic characteristics and the time course of primary PPV, duration of SO in situ, and axial length (AL) as measured by an IOL master (Carl Zeiss IOL Master, Germany) before SO removal were also collected from medical records.

### 2.2. Particle Collection and Measurement

Using the method reported by Chan et al. [9], we collected the first 2 mL of washout fluid after the SO was removed, and using a Multisizer® 3 Coulter counter, (Beckman Coulter, USA) the size and number of droplets in the washout were measured. The Multisizer® simultaneously counts and measures the size of individual particles, and to ensure our method was comparable with that used by Chan et al. [9], this time particles ranging in diameter from 1 to 12 *μ*m were measured. The values for each sample represent the mean of three consecutive measurements. In the study, we use a measuring probe with a small aperture hole, 20 *μ*m in diameter. In order to control the coincidence effect under 5%, according to the international standard ISO 13319, the counts per milliliter should be between 5000 and 250,000. At the beginning, we test 3 samples and diluted 20 times. The mean number of particles was 3.25 × 10^6^/ml. As a result, during the study, all samples were diluted 50 times before measurement. In that case, the number of droplets should be around 6 × 10^4^/ml, within the optimal range.

### 2.3. Statistical Analysis

The mean and standard deviations were calculated for SO droplets and for other clinical variables. Correlations of the size and number of SO droplets with age, IOP, duration of SO in situ, and time-course of primary PPV were determined using Spearman's correlation coefficient. Multiple regression analysis was then used to determine which parameters were significantly correlated with SO droplet size and number; statistical significance was defined as *P* < 0.05.

We also divided the patients into three groups according to their use of antiglaucoma medications before and 3 months after SO removal. The Mann–Whitney *U* test was used for comparisons between pairs of groups, with statistical significance set at *P* < 0.05. All analyses were performed using SPSS software version 20.0 (SPSS, Inc., Chicago, IL, USA).

## 3. Results

A total of 38 patients (23 males, 15 females) who underwent primary PPV with SO injection for RRD and whose retina stayed attached for ≥3 months after SO removal were included in the study. Only one type of SO (5700 cSt; Bausch and Lomb Inc., USA) was used in these patients. Their mean age was 56 ± 11 years (range 20–70 years), mean AL was 26.0 ± 2.6 mm (range 22.2–33.1 mm), mean duration of SO tamponade was 192 ± 97 days (range 83–680 days), and mean operation time for primary PPV was 70 ± 16 min (range 45–105 min) ([Supplementary-material supplementary-material-1], Table 1). At the time of SO removal, 17 patients were using topical antiglaucoma medications, and in 7 of these patients, IOP was higher than 21 mmHg (mean 25.8 mmHg, range 22–37 mmHg). After SO removal, eight patients were still using antiglaucoma eye drops at their last visit, with IOP <21 mmHg in all of these patients. At the time of primary PPV, 5 patients were pseudophakic and 33 were phakic; in 26 of these 33 patients, their crystal lens was removed by phacoemulsification during PPV.

**Figure 1:**
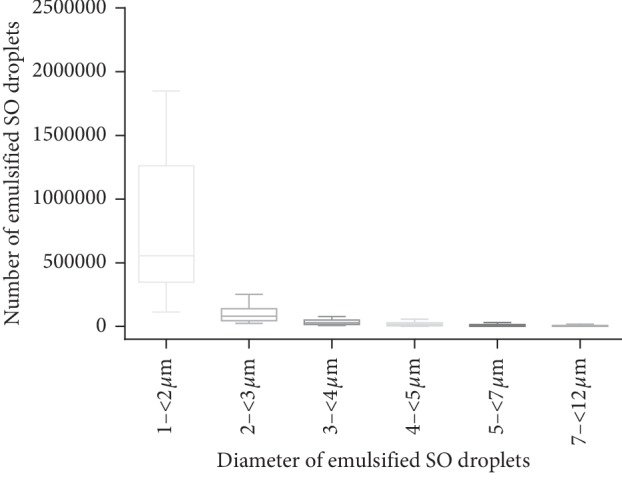
The size distribution of silicone oil droplets in box and whisker plots.

The size and number of emulsified SO droplets were successfully measured in samples from all of the patients. The mean number of droplets found in the washout samples was 1.96 × 10^6^ ± 3.95 × 10^6^/ml (range 0.17 × 10^6^–21.71 × 10^6^/ml). The size distribution of particles is listed in Table 1 and Figure 1; overall, 80.8% (range 64.23%–99.07%) of the droplets were 1-2 *μ*m in diameter.

The statistical tests did not reveal any correlation between the total number of emulsified SO droplets and any of the clinical factors (all *P* > 0.05, Table 2). However, when the emulsified SO droplets were divided into groups by their diameter, multiple linear regression revealed that age was negatively correlated with the number of 5–7 *μ*m-diameter and 7–12 *μ*m-diameter droplets (both *P* < 0.05, Table 2).

**Figure 2:**
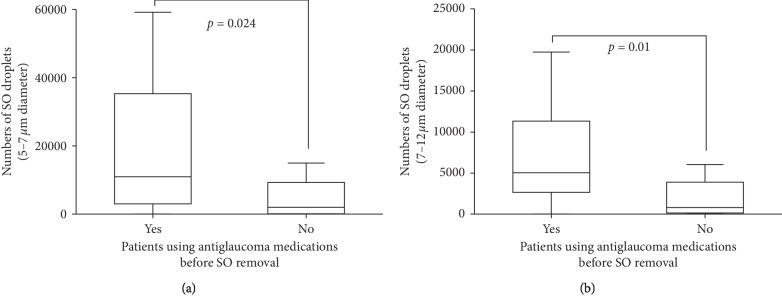
Box and whisker plots comparing the numbers of 5–7 *μ*m-diameter (a) and 7–12 *μ*m-diameter (b) silicone oil droplets between patients using antiglaucoma medications and patients not using antiglaucoma medications at the time of silicone oil removal.

**Figure 3:**
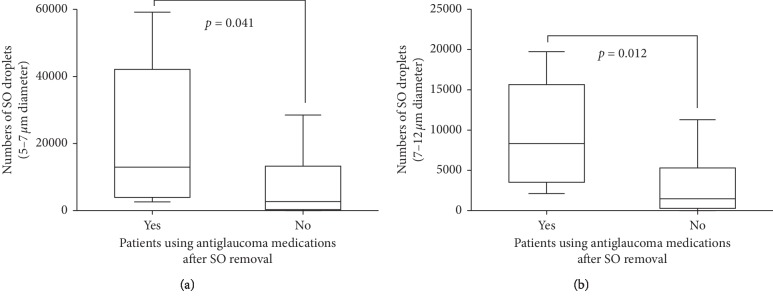
Box and whisker plots comparing the numbers of 5–7 *μ*m-diameter (a) and 7–12 *μ*m-diameter (b) silicone oil droplets between patients using antiglaucoma medications and patients not using antiglaucoma medications after SO removal.

Next, we divided the patients into different groups according to their use of antiglaucoma medications. Before SO removal, patients who were using antiglaucoma medications were similar in terms of patient age, the duration of SO in situ, time-course of primary PPV, AL, and total number of emulsified droplets as patients who were not using antiglaucoma medications. However they had significantly more 5–7 *μ*m-diameter and 7–12 *μ*m-diameter droplets (all *P* < 0.05, Figure 2, Table 3). After silicone removal, patients using antiglaucoma medications still demonstrated more 5–7 *μ*m-diameter and 7–12 *μ*m-diameter droplets than those not using (all *P* < 0.05, Figure 3, Table 4), but similar other factors (all *P* > 0.05, Table 4).

## 4. Discussion

In this study, we successfully measured both the size and number of emulsified SO droplets for the first time in a large group of patients. The mean number of droplets found in the washout samples was 1.96 × 10^6^ ± 3.95 × 10^6^/ml, and 80.8% of the droplets were 1-2 *μ*m in diameter. The size distribution of the SO droplets observed in this study was almost identical to that reported by Chan et al. [9] ([Supplementary-material supplementary-material-1], Table 1). These suggested that the emulsification of SO we saw in the clinic was just the tip of the iceberg, and most of the emulsified SO droplets are not visible under a microscope.

We also investigated whether the number and size of SO droplets were correlated with clinical characteristics. Although the number of total SO droplets was not correlated with any of the clinical characteristics, the number of 5–12 *μ*m-diameter droplets was significantly and negatively correlated with patient age. The reason for this finding is not fully clear, and some of the following factors might contribute to it. An earlier study revealed that many substances, including proteins and phospholipids, could accelerate SO emulsification by reducing the surface tension of the SO. [14–16] Savion et al. [15] also reported that red blood cell membranes and plasma lipoproteins promoted SO emulsification. It was reported that exudation was the most common complication after vitreoretinal surgery [17]. Oshika [18] found that the aqueous protein concentration increased after scleral buckling procedures and was closely correlated with patient age. Therefore, younger patients, more severe postoperative exudation, and higher concentrations of proteins or other substances could accelerate emulsification. It is also reported that shearing forces at the interface between SO and aqueous layers could affect emulsification [19]. Increased and pronounced eye movements were found to facilitate SO emulsification [20]. Because younger patients might be more active, this could be another potential explanation for the greater emulsification in these patients. Furthermore, a relative underfill of oil may occur in younger patients and in phakic patients in whom less vitreous is generally removed leading to more residual aqueous in the system, hence more propensity to emulsify.

We also found that patients who used antiglaucoma medications before or after SO removal had more 5–7 *μ*m-diameter and 7–12 *μ*m-diameter droplets than patients who did not use antiglaucoma medications. On the other hand, after SO removal, the number of patients who required topical antiglaucoma medication decreased significantly from 17 to 8 (*P*=0.002). Even in patients who were still using antiglaucoma medications, the number of eyedrops decreased from 2 to 1.25 (*P*=0.02). This might suggest a relationship between SO emulsification and elevated IOP, but only for larger SO droplets. The size of the intertrabecular spaces in the trabecular network might contribute to this finding. The intertrabecular spaces of the scleral meshwork are ovals, with greater and lesser axes of 10 *μ*m and 5 *μ*m, respectively, [21] and this is in accordance with and could explain our findings that patients with much more droplets of 5–12 *μ*m-diameter were more likely to be using antiglaucoma medications.

The results of the study by Chan et al. [9] and our study indicate that most of the emulsified SO droplets were 1-2 *μ*m in diameter, and SO emulsification is likely to be underestimated in the clinic when assessed using the traditional methods. Considering that all of the patients in our study received SO with a viscosity of 5700 cSt, it is possible that the extent of SO emulsification could be more severe when using lower-viscosity SO, especially in younger patients. Therefore, we believe that, to limit the extent of SO emulsification, SO should be removed once the retinal tear has sealed and the PVR seems to be stationary.

Our study was limited by its cross-sectional design, the limited number of patients, and the fact that only one kind of SO and only Chinese patients who underwent surgery by one surgeon were included.

## 5. Conclusion

We showed that, using a Multisizer® Coulter counter, it is possible to evaluate the extent of SO emulsification following PPV. The number of 5–12 *μ*m-diameter droplets was higher in younger-age patients and was higher in patients using antiglaucoma eyedrops. Future studies in this field were still required to improve our knowledge further.

## Figures and Tables

**Table 1:** Size distribution of silicone oil droplets.

Diameter	Number of droplets	Percentage, mean ± SD
1-<2 *μ*m	1.76 × 10^6^ ± 3.91 × 10^6^	80.80% ± 8.93%
2-<3 *μ*m	1.00 × 10^5^ ± 0.64 × 10^5^	9.88% ± 3.67%
3-<4 *μ*m	4.76 × 10^4^ ± 8.25 × 10^4^	4.42% ± 5.16%
4-<5 *μ*m	3.58 × 10^4^ ± 7.99 × 10^4^	2.98% ± 4.85%
5–<7 *μ*m	1.25 × 10^4^ ± 1.66 × 10^4^	2.23% ± 3.10%
7–<12 *μ*m	0.52 × 10^4^ ± 0.60 × 10^4^	1.03% ± 1.56%

Values are expressed as mean ± standard deviation.

**Table 2:** Associations between clinical characteristics and size and number of silicone oil droplets.

Clinical characteristic	Number and diameter of SO droplets						
	Total number	1-<2 *μ*m	2-<3 *μ*m	3-<4 *μ*m	4-<5 *μ*m	5–<7 *μ*m	7–12 *μ*m
Age	NS	NS	NS	NS	NS	*β* = −0.44 *P*=0.006	*β* = −0.38 *P*=0.016
AL	NS	NS	NS	NS	NS	NS	NS
IOP	NS	NS	NS	NS	NS	NS	NS
Duration of SO in situ	NS	NS	NS	NS	NS	NS	NS
Time course of PPV	NS	NS	NS	NS	NS	NS	NS

Multiple linear regression was used to assess correlations between clinical characteristics and SO characteristics. NS, not significant; AL, axial length; IOP, intraocular pressure; SO, silicone oil; PPV, pars plana vitrectomy.

**Table 3:** Comparison of clinical factors and characteristics of emulsified silicone oil droplets between patients using or not using antiglaucoma medications at the time of silicone oil removal.

Variable	Use of antiglaucoma medications		*P*
	Yes	No	
Patients	17	21	
Age (years)	55 ± 13	56 ± 9	1.000
AL (mm)	25.32 ± 2.16	26.57 ± 2.80	0.189
IOP (mmHg)	21.4 ± 59	15.6 ± 2.8	0.000^*∗*^
Duration of SO in situ (days)	179 ± 52	201 ± 122	0.794
Time course of vitrectomy (min)	70 ± 17	70 ± 15	0.862
Total number of droplets	0.96 × 10^6^ ± 0.77 × 10^6^	2.78 × 10^6^ ± 5.18 × 10^6^	0.586
Droplets in different diameter			
1-<2 *μ*m	0.73 × 10^6^ ± 0.67 × 10^6^	2.59 × 10^6^ ± 5.13 × 10^6^	0.243
2-<3 *μ*m	1.00 × 10^5^ ± 0.71 × 10^5^	1.00 × 10^5^ ± 0.60 × 10^5^	0.772
3-<4 *μ*m	6.37 × 10^4^ ± 11.90 × 10^4^	3.45 × 10^4^ ± 2.95 × 10^4^	0.794
4-<5 *μ*m	4.30 × 10^4^ ± 11.15 × 10^4^	3.00 × 10^4^ ± 4.23 × 10^4^	0.523
5–<7 *μ*m	1.76 × 10^4^ ± 1.81 × 10^4^	0.84 × 10^4^ ± 1.43 × 10^4^	0.024^*∗*^
7–<12 *μ*m	0.71 × 10^4^ ± 0.58 × 10^4^	0.35 × 10^4^ ± 0.58 × 10^4^	0.010^*∗*^

Values are expressed as the mean ± standard deviation. ^*∗*^*P* < 0.05 was considered statistically significant. AL, axial length; IOP, intraocular pressure; SO, silicone oil; PPV, pars plana vitrectomy.

**Table 4:** Comparison of clinical factors and characteristics of emulsified silicone oil droplets between patients using or not using antiglaucoma medications after silicone oil removal.

Variable	Use of antiglaucoma medications		*P*
	Yes	No	
Patients	8	30	
Age (years)	54 ± 15	56 ± 9	0.792
AL (mm)	25.36 ± 2.79	26.18 ± 2.540	0.492
IOP (mmHg)	21.1 ± 5.1	17.4 ± 5.1	0.028^*∗*^
Duration of SO in situ (days)	163 ± 44	199 ± 106	0.297
Time course of vitrectomy (min)	70 ± 16	70 ± 16	1.000
Total number of droplets	0.84 × 10^6^ ± 0.54 × 10^6^	2.26 × 10^6^ ± 4.40 × 10^6^	0.586
Droplets in different diameter			
1-<2 *μ*m	0.65 × 10^6^ ± 0.43 × 10^6^	2.06 × 10^6^ ± 4.35 × 10^6^	0.515
2-<3 *μ*m	1.02 × 10^5^ ± 0.72 × 10^5^	1.00 × 10^5^ ± 0.63 × 10^5^	1.000
3-<4 *μ*m	4.17 × 10^4^ ± 3.58 × 10^4^	4.92 × 10^4^ ± 9.15 × 10^4^	0.847
4-<5 *μ*m	1.79 × 10^4^ ± 1.44 × 10^4^	4.06 × 10^4^ ± 8.94 × 10^4^	0.739
5–<7 *μ*m	2.18 × 10^4^ ± 2.14 × 10^4^	1.00 × 10^4^ ± 1.45 × 10^4^	0.041^*∗*^
7–<12 *μ*m	0.95 × 10^4^ ± 0.64 × 10^4^	0.40 × 10^4^ ± 0.54 × 10^4^	0.012^*∗*^

Values are expressed as mean ± standard deviation. ^*∗*^*P* < 0.05 was considered statistically significant. AL, axial length; IOP, intraocular pressure; SO, silicone oil; PPV, pars plana vitrectomy.

## Data Availability

The research data used to support the findings of this study are included within the article.

## Supplementary Materials

Supplementary Materials Table S1: the clinical characteristics of the patients. Figure S1: size distribution of emulsified silicone oil droplets reported in our study and in the study by Chan et al.
