# Supplementary material for: Silicone Oil Emulsification after Vitrectomy for Rhegmatogenous Retinal Detachment
Source: J Ophthalmol. 2020 Feb 24;2020:6940625. doi: 10.1155/2020/6940625 (PMC7060885; doi:10.1155/2020/6940625)
Supplement: Supplementary Materials — Table S1: the clinical characteristics of the patients. Figure S1: size distribution of emulsified silicone oil droplets reported in our study and in the study by Chan et al. [file 6940625.f1.docx]

**Table S1.** Patient characteristics

| **Variable** | **Value** |
| --- | --- |
| Number | 38 |
| Sex (male/female) | 23/15 |
| Age (years) | 56 ± 11 (20–76) |
| AL (mm) | 26.0 ± 2.6 (22.2–33.1) |
| IOP (mmHg) | 18.2 ± 5.3 (10–37) |
| mean duration of SO in situ (days) | 192 ± 97 (83–680) |
| Mean operation time of vitrectomy (min) | 70 ± 16 (45–105) |
| Lens state at the time of primary PPV (phakic/pseudophakic) | 33/5 |
| Ccombined cataract surgery (yes/no) | 26/7 |
| Number of emulsified droplets | 1.96 × 10^6^ ± 3.95 × 10^6^ (0.17 × 10^6^–21.17 × 10^6^) |
| Patients using anti-glaucoma drugs at the time of SO removal | 17 |
| Patients using antiglaucoma drugs after SO removal | 8 |

Values are expressed as the number of patients or as the mean ± standard deviation (range).

AL, axial length; IOP, intraocular pressure; SO, silicone oil; PPV, pars plana vitrectomy

**Figure S1.** Size distribution of emulsified silicone oil droplets reported in our study and in the study by Chan et al^9^.
